# Clinical experience with sodium–glucose transport 2 (SGLT2) inhibitors in patients with systemic lupus erythematosus

**DOI:** 10.1007/s11255-025-04815-5

**Published:** 2025-10-17

**Authors:** Oshrat E. Tayer-Shifman, Irina Kenis, Sydney Benchetrit, Dorin Bar-Ziv, Yael Pri-Paz Basson, Yair Levy, Shaye Kivity, Keren Cohen-Hagai

**Affiliations:** 1 Rheumatology Unit, Meir Medical Center, Kfar Saba, Israel; 2 Faculty of Medical and Health Sciences, Tel-Aviv University, Tel-Aviv, Israel; 3 Department of Nephrology and Hypertension, Meir Medical Center, Kfar Saba, Israel; 4 Department of Internal Medicine B, Meir Medical Center, Kfar Saba, Israel; 5 Department of Internal Medicine E, Meir Medical Center, Kfar Saba, Israel

**Keywords:** Systemic lupus erythematosus, Lupus nephritis, Chronic kidney disease, Non-immunosuppressive treatment, Sodium–glucose transport 2 inhibitors

## Abstract

**Objectives:**

Effective management of chronic kidney disease (CKD) in systemic lupus erythematosus (SLE) is crucial for reducing long-term complications. Sodium–glucose transport 2 inhibitors (SGLT2is) improve renal and cardiovascular outcomes of non-SLE-related CKD. We aimed to assess clinical characteristics and outcomes of SLE patients treated with SGLT2is.

**Methods:**

This retrospective study evaluated adult SLE patients treated with SGLT2is for ≥ 3 months compared to untreated patients. Disease-related characteristics, kidney-related factors, and comorbidities were assessed. Repeated measures analysis model was applied to compare estimated glomerular filtration rate (eGFR) at different timepoints. Additional outcomes included infections, hospitalizations, and all-cause mortality. In SGLT2is-treated patients, eGFR and albuminuria were recorded before SGLT2is initiation, at 1 month, and 1 year following SGLT2is initiation.

**Results:**

A total of 112 consecutive patients were included with a mean follow-up of 18.1 ± 12.7 years since SLE diagnosis. The use of SGLT2is in 9 SLE patients showed a stable kidney function over-time (60 ml/min/m^2^ ± 15 before treatment vs. 60 ± 17 ml/min/m^2^ at 1 year of treatment, p = 0.5) and a trend toward reduction in proteinuria (450 ± 637 mg/g before treatment to 279 ± 401 mg/g at 12 months following treatment, p = 0.07), without major adverse effects. No significant differences in cardiovascular events, infections, hospitalizations, or mortality rates were observed between treated and untreated patients.

**Conclusions:**

This study suggests a potential benefit of SGLT2is in SLE patients. Further research is needed to confirm the nephro- and cardioprotective effects of SGLT2is in this population.

## Introduction

Renal involvement is common in systemic lupus erythematosus (SLE), with lupus nephritis (LN) occurring in up to 50% of patients [1]. Patients who had LN are regarded as having chronic kidney disease (CKD), since kidney damage and nephron loss occur very early during the course of LN, as shown in repeat kidney biopsies [2]. Kidney scarring occurs despite early immunosuppressive treatment, even in patients who reach clinical renal response with preserved kidney function and no residual proteinuria [3]. Hyperfiltration by the remaining functional nephrons accelerates kidney function loss and increases proteinuria, independent of immunological activity [2].

Around 10% of patients with LN will develop end-stage kidney disease (ESKD), eventually needing kidney replacement therapy [1]. Nonetheless, the complications of CKD in SLE patients extend beyond the kidneys, leading to an increased risk of hypertension, cardiovascular morbidity and mortality, increased infection rates, and all-cause mortality [4–10]. Effective management of LN and CKD is essential to mitigate the risk of complications and optimize long-term outcomes and survival for patients with SLE [7].

Treatment options in patients with SLE and kidney disease include both immunosuppressive and non-immunosuppressive therapies. While extensive research has focused on immunosuppressive therapies, establishing them as pivotal in the management of lupus nephritis, non-immunosuppressive therapies such as renin–angiotensin–aldosterone system (RAAS) blockade, salt and protein-restricted diet, blood pressure control, and lipid-lowering drugs are generally recommended but less studied [11–16].

In recent years, there has been a growing awareness of the importance of strategies addressing non-immunosuppressive measures, including kidney-protective agents for managing CKD. Kidney-protective drugs with sodium–glucose transport 2 inhibitors (SGLT2is) combined with RAAS blockade improved kidney outcomes in CKD of various causes, and improved cardiovascular outcomes and/or all-cause death in diabetic kidney disease or non-diabetic CKD [17–19].

One of the mechanisms that may explain the benefits of SGLT2is on kidney disease progression is their effect on tubule–glomerular feedback, resulting in a reduction of glomerular hyperfiltration, which complements the effect of RAAS blockade [20]. Hence, it was suggested that SGLT2is may reduce proteinuria and improve kidney outcomes in non-diabetic patients as well [17, 18, 21–24]. Due to the common final pathway of CKD, and the increased cardiovascular and renal risk of SLE patients, it was proposed that, likewise, SGLT2is may have a reno- and cardioprotective effect in patients with SLE as well, as evidenced by a multi-center cohort study demonstrating that treatment with SGLT2is resulted in a significantly lower risk of new-onset acute kidney injury, chronic kidney disease, end-stage renal disease, and heart failure among patients with SLE [25]. Moreover, specific complications of SLE may also benefit from the therapeutic effect of SGLT2is, including increased risk for diabetes, hypertension, and metabolic syndrome [7, 18, 22, 23].

Recently, the EULAR, ACR, and the KDIGO lupus nephritis guidelines updated their recommendations for the management of lupus nephritis, suggesting the consideration of SGLT2is as kidney-protective agents for stable patients with CKD, diabetes mellitus (DM), moderate–high proteinuria, or heart failure on RAAS blockade [12, 13, 26]. Despite these recommendations, there is a scarcity of studies that have specifically explored the efficacy and safety of SGLT2is among individuals with SLE [21]. This highlights the need for further research to better understand the potential benefits and risks of incorporating SGLT2is into the treatment regimens for patients with SLE.

This study aims to assess the clinical characteristics and outcomes of Israeli patients with SLE treated with SLGT2is, with a focus on selected comorbidities, such as CKD, diabetes mellitus, and heart failure.

## Methods

### Study design

This retrospective observational study, conducted at Meir Medical Center, between 2014 and 2024, included adult patients with SLE diagnosed for at least 12 months, with follow-up until 1.1.2024.

Results are reported according to the STROBE statement guidelines.

### *Study population* and measured outcomes

Patients receiving SGLT2is for a duration of 3 months or longer were compared with a control group consisting of SLE patients who were not treated with these agents. Patients < 18 years of age or missing electronic medical records (EMR) data were excluded.

Associated variables were assessed, including disease-related characteristics, kidney-related factors, and comorbidities, such as diabetes and heart failure. Kidney function was assessed using estimated GFR (eGFR) based on the CKD epidemiology collaboration equation [22] at different timepoints before and after SLE diagnosis. eGFR was calculated for each patient, 1 year before SLE diagnosis (“before diagnosis”), at SLE diagnosis, 2 and 5 years following SLE diagnosis, and at the last follow-up, which was defined as the final available follow-up measurement for each patient, and, therefore, varied in timing across individuals. In SGLT2is-treated patients, serum creatinine and eGFR were specifically recorded before SGLT2is initiation, 1 month, and 1 year following SGLT2is initiation. Due to the retrospective nature of the data set, usage rates of RAAS inhibitors were available only in the SGLT2is-treated group.

Additional clinical outcomes, including infections, hospitalizations, and all-cause mortality were recorded. Admission and discharge diagnoses were recorded by the attending physicians based on clinical, electrocardiographic, and biochemical criteria. Mortality data were determined from electronic medical records (EMR) and by the national registry.

### Ethical considerations

The study was approved by the Institutional Ethics Committee in keeping with the principles of the Declaration of Helsinki. In accordance with the Ministry of Health regulations, the Institutional Ethics Committee did not require written informed consent, since data were collected anonymously from the EMR without active patient participation.

### Statistical analysis

Data are presented as numbers and percentages for nominal parameters and as means and standard deviations for continuous parameters. Differences between the study groups were analyzed with chi-square test. *T* test or one-way ANOVA was used for normally distributed variables. Analyses were performed using available data only, with missing values (e.g., follow-up eGFR and albuminuria) excluded from the respective analyses.

Repeated measures analysis model was applied to compare the eGFR at different timepoints (before diagnosis, at diagnosis, and the last follow-up), among the SGLT2is-treated patients and controls. Protein to creatinine ratio was compared before starting treatment with SGLT2is and on treatment at the last follow-up. Mortality rate between groups was compared with Kaplan–Meier survival curve, stratified by SGLT2is use. P < 0.05 was considered statistically significant. Data were analyzed with SPSS Version 27 (IBM Corporation, Armonk, NY).

## Results

A total of 112 consecutive patients were included in this analysis. Table 1 provides the demographics and clinical characteristics of the cohort.

**Table 1:** Demographics and clinical characteristics of patients treated and not treated with SGLT2is

	SGLT2is-treated patients *N* = 9	Controls *N* = 103	*p* value
Female, *n* (%)	6 (66.7%)	88 (85.4%)	0.141
Age at diagnosis, years ± SD	36.1 ± 14.3	33.5 ± 14.9	0.607
Age at last follow-up	60.11 ± 15.2	50.4 ± 16.0	0.082
Follow-up time, years ± SD	25.1 ± 12.1	17.5 ± 12.8	0.088
Ethnicity, n (%)			
Jews	7 (77.8%)	74 (71.8%)	0.817
Arabs	2 (22.2%)	25 (24.3%)	
Other	0 (0.0%)	4 (3.9%)	
Lupus nephritis	4 (44.4%)	42 (40.8%)	0.830
Class III/IV in kidney biopsy	4 (44.4%)	29 (28.1%)	0.304
eGFR at SLE diagnosis	73 ± 47	88 ± 32	0.4
Comorbidities, n (%)			
IHD	4 (44.4%)	10 (9.7%)	**0.003**
CHF	3 (33.3%)	6 (5.8%)	**0.004**
Diabetes mellitus	6 (66.7%)	9 (8.7%)	** < 0.0001**
Hypertension	6 (66.7%)	43 (41.7%)	0.133
CVA	0 (0.0%)	10 (9.7%)	0.327
APS	0 (0.0%)	31 (30.1%)	0.053
FMS	0 (0.0%)	7 (6.8%)	0.419
Last SBP	146.8 ± 22.6	125.7 ± 19.3	**0.002**
Last DBP	78.1 ± 18.4	74.1 ± 11.9	0.354
SDI score	3.3 ± 2.7	2.6 ± 2.6	0.394
Medications^a^			
GC ever	9 (100%)	78 (85.7%)	0.518
HCQ ever	88 (96.7%)	8 (88.9%)	0.254
AZA ever	33 (36.3%)	1 (12.5%)	0.391
MMF ever	4 (50.0%)	37 (40.7%)	0.792
CYC ever	3 (37.5%)	20 (22.0%)	0.447
BEL ever	2 (22.2%)	26 (28.6%)	0.246
RTX ever	0 (0.0%)	7 (7.7%)	0.613

*Bold values indicate statistical significance at p* < *0.05*

^**a**^Medications data available only for 100 patients (9 of the SGLT2i-treated group and 91 of the controls)

*SGLT2i* sodium–glucose transport 2 inhibitors, *SLE* systemic lupus erythematosus, *IHD* ischemic heart disease, *CHF* congestive heart failure, *CVA* cerebrovascular accident, *ESKD* end-stage kidney disease, *APS* antiphospholipid syndrome, *FMS* fibromyalgia, *SBP* systolic blood pressure, *DBP* diastolic blood pressure, *SDI* Systemic Lupus International Collaborating Clinics /American College of Rheumatology Damage Index, *GC* glucocorticoids, *HCQ* hydroxychloroquine, *AZA* azathioprine, *MMF* mycophenolate mofetil, CYC cyclophosphamide, *BEL* belimumab, *RTX* rituximab

Only 6 out of 15 patients with diabetes (40%), 3 out of 9 patients with heart failure (33.3%), and 7 out of 51 patients with residual proteinuria on the last follow-up (13.7%) were treated with SGLT2is.

### Characteristics of SGLT2is-treated patients

Nine out of 112 were treated chronically with SGLT2is and had available data (Table 2). Of them, 6 were females (66.7%), the mean age at SLE diagnosis was 36.1 years (range 21.2–56.1), and the mean age at last follow-up was 62.4 years (range 34.4–85.6). The mean SGLT2is treatment duration was 21.2 months (range 3–75.9), and this treatment was initiated 24.3 ± 10.8 years following SLE diagnosis. Five patients were treated with dapagliflozin, 2 with empagliflozin, and 2 switched from empagliflozin to dapagliflozin (one due to administrative reasons and one due to side effects). Indications for SGLT2is included CKD [6 patients], DM [2], and CHF [1]. Six patients (66.7%) had a history of LN. Comorbidities included DM [6 patients], hypertension [6], and ischemic heart disease [4]. All patients received concomitant RAAS blockade, except for one (due to hyperkalemia).

**Table 2:** Clinical characteristics of SGLT2is-treated SLE patients

	1	2	3	4	5	6	7	8	9
Sex, Age at Diagnosis (years)	F, 56.1	F, 38.3	F, 52.9	F, 16.8	F, 22.8	F, 48.9	M, 35.0	M, 21.2	M, 33.3
Age at last follow-up	85.6	70.6	62.5	34.4	62.7	60.0	60.6	47.4	77.6
Ethnicity	Jewish	Arab	Jewish	Jewish	Jewish	Jewish	Jewish	Arab	Jewish
Main SGLT2i indication	CHF	CKD	CKD	CKD	CKD	CKD	CKD	DM	DM
SGLT2i type	Empagliflozin Dapagliflozin	Empagliflozin	Dapagliflozin	Dapagliflozin	Empagliflozin	Dapagliflozin	Dapagliflozin	Dapagliflozin	Empagliflozin Dapagliflozin
Side effects	Diarrhea	–	–	–	Simple UTI	Simple UTI	–	–	–
Treatment continuity	Stopped	+	+	+	+	+	+	+	+
SGLT2i duration (months)	4.1	15.8	3.0	4.5	30.8	30.9	14.0	12.3	75.9
Urinary albumin before, at 12 months (µg/l)	24, 0	349, 62	91, NA	823, NA	425, 409	166, 0	1851, 1116	26, 61	311, 304
SLE manifestations	Skin, joints, PLT↓, anti-dsDNA +	Skin, joints, LN, anti-dsDNA + , C3/C4↓	Skin, LN, PLT↓, anti-dsDNA + , C3/C4↓, Class III/IV LN	Skin, joints, LN, anti-dsDNA + , C3/C4↓ Class III/IV LN	Skin, joints, LN, anti-dsDNA +	Skin, joints, LN, anti-dsDNA + , C3/C4↓ Class III/IV LN	Fever, Skin, PLT↓, anti-dsDNA + , C3/C4↓	joints, pericarditis, LN, seizures, anti-dsDNA + , C3/C4↓ Class III/IV LN	Joints, anti-dsDNA +
Comorbidities	DM, HTN, CHF, IHD	DM, HTN, CHF, IHD	HTN	HTN	DM	DM, HTN	HTN	DM, IHD	DM, CHF. IHD
SLE medications	-	HCQ	HCQ, MMF	HCQ, GC, MMF, BEL	HCQ	HCQ, GC, MMF, BEL	HCQ	HCQ, GC MMF	HCQ
Other medications	ACE-I, MRA, Statin, SSRI, diuretics, BB, AC, PPI, EPO	ARB, MRA, statin, diuretics, ASA, BB, CCB, AC	ARB, statin	ACE-I, CCB, BB, statin, PPI	ACE-I, ASA, statin	ACE-I, BB, CCB, PPI	CCB, BB, AC	ACE-I, statin, fibrate, BB, ASA, AC, PPI, metformin	ARB, statin, BB, CBB, diuretics, ASA, insulin, SSRI
SDI score	7	5	1	0	3	0	4	3	7
Mortality	–	–	–	–	–	–	–	Deceased	–

*SGLT2i* sodium–glucose transport 2 inhibitors, *SLE* systemic lupus erythematosus, *SDI* Systemic Lupus International Collaborating Clinics /American College of Rheumatology Damage Index, *CHF* congestive heart failure, *CKD* chronic kidney disease, *DM* type 2 diabetes mellitus, *UTI* urinary tract infection, *PLT* platelets, *dsDNA* double-stranded DNA, *LN* lupus nephritis, *HTN* hypertension, *IHD* ischemic heart disease, *HCQ* hydroxychloroquine, *MMF* mycophenolate mofetil, *GC* glucocorticoids (prednisone 5 mg/day), *BEL* belimumab, *ACE-I* angiotensin-converting enzyme inhibitor, *MRA* mineralocorticoid receptor antagonist, *SSRI* selective serotonin reuptake inhibitor, *BB* beta-blocker, *AC* anti-coagulation. *PPI* proton-pump inhibitor, *EPO* erythropoietin, *ARB* angiotensin receptor blocker, *ASA* acetylsalicylic acid or different anti-platelet, *CCB* calcium-channel blockers

**Fig. 1:**
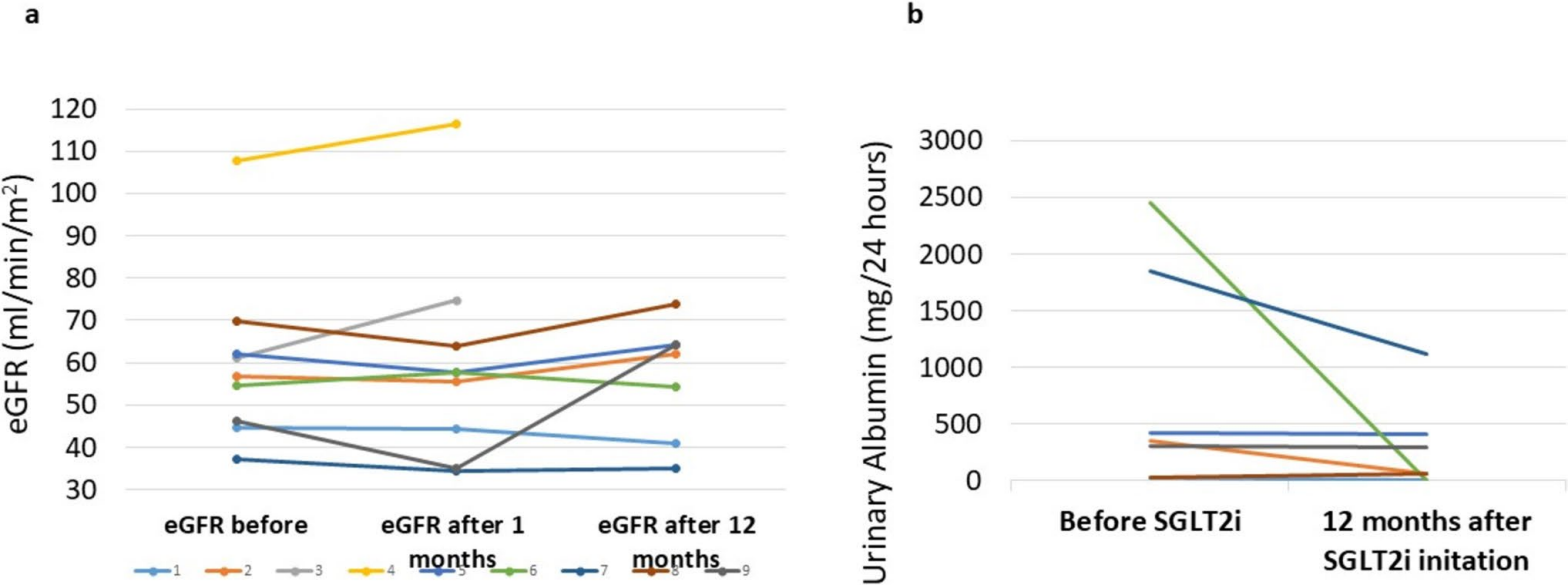
Kidney function in SGLT2is-treated SLE patients before and after treatment including (a) Estimated glomerular filtration rate (eGFR) before, at 1 month of treatment, and 12 months of treatment; and (b) urinary albumin (mg/24 h) before treatment and at 12 months of treatment. *eGFR* estimated glomerular filtration rate, *SGLT2i* sodium–glucose transport 2 inhibitors

Among 7 patients treated with SGLT2is ≥ 12 months, mean urine albumin decreased from 450 ± 637 mg/g before treatment to 279 ± 401 mg/g at 12 months following treatment (p = 0.07). As expected, we observed a slight increase in serum creatinine after 1 month of treatment (from 1.23 mg/dl, range 0.92–1.87 to 1.32 mg/dl, range 0.98–1.99), reflecting the dip phenomenon, while during longer follow-up, eGFR remained stable (60 ± 15 to 60 ± 17 ml/min/m^2^, respectively, p = 0.5). (Fig. 1).

There was 1 death due to disseminated zoster infection. Two patients had an episode of uncomplicated urinary tract infection (UTI), and one patient discontinued SGLT2is treatment (empagliflozin and dapagliflozin) due to diarrhea. No patient developed end-stage kidney disease (ESKD) or new cardiovascular events during the study period. Two patients had hospitalizations during follow-up, one with disseminated zoster and the other with 4 hospitalizations due to biliary pancreatitis [2], diabetic foot, and syncope.

### Renal outcomes

No patients treated with SGLT2is had developed ESKD, while 13 patients (12.6%) in the control group developed ESKD during the study period. Patients with ESKD were excluded from the rest of the analysis of renal outcomes, including eGFR and albuminuria.

**Fig. 2:**
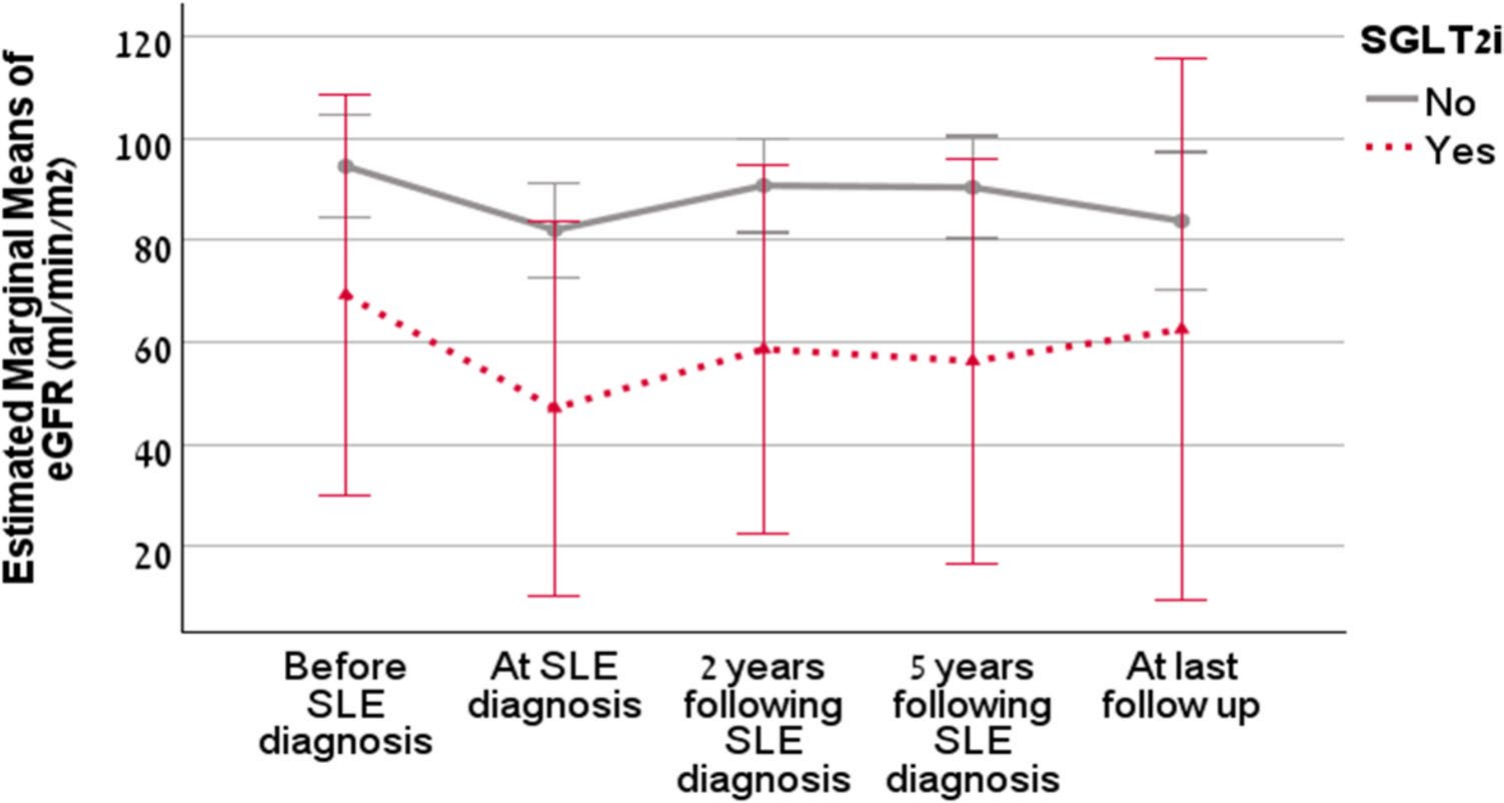
Repeated measure analysis for estimated glomerular filtration rate (eGFR) at different timepoints with 95% confidence intervals among patients treated with SGLT2is compared to untreated patients, *before diagnosis* is defined as 1 year prior to SLE diagnosis and *last visit* denotes the final available follow-up for each patient with variable timing across individuals. *Time from SLE diagnosis to SGLT2i initiation varied among treated patients.* *SLE* systemic lupus erythematosus, *eGFR* estimated glomerular filtration rate, *SGLT2i* sodium–glucose transport 2 inhibitors

On repeated measures analysis, eGFR decreased significantly during the study period from a mean of 92.5 before diagnosis to 88.3 ml/min/m^2^ at SLE diagnosis and to 96.8 ± 34.9, 96.1 ± 38.2, and 76.8 ± 41.9 at 2, 5, and last follow-up, respectively (p > 0.01). Figure 2 shows eGFR trends of SGLT2is-treated patients compared to controls over five timepoints. SGLT2is-treated patients had lower GFR at baseline and diagnosis; however, while the eGFR of controls declined over-time, the eGFR of SGLT2is-treated patients stabilized. There was a trend toward longer follow-up duration in the SGLT2is-treated group compared with controls, and the time from SLE diagnosis to SGLT2i initiation also varied among treated patients. The 95% confidence intervals for the two groups overlapped at all timepoints, and the intervals for the SGLT2is-treated group were markedly wider, reflecting the small number of patients and greater variability in this group. These factors suggest cautious interpretation.

### Cardiovascular and other outcomes

Hospitalization for cardiovascular events was comparable between groups (22.2% in SGLT2is-treated patients vs. 15.7% in controls, p = 0.6), as well as hospitalizations due to severe infections (22.2% vs. 30.3%, p = 0.6) or SLE exacerbation (33.3% vs. 24.7%, p = 0.6) (Table 3).

**Table 3:** Outcomes of patients treated and untreated with SGLT2 inhibitors

Outcome	SGLT2i-treated patients *N* = 9	Controls *N* = 103	*p* value
ED visits	4.2 ± 2.9	6.0 ± 7.5	0.475
Hospitalizations, all-cause	5 (55.6%)	48 (53.9%)	0.924
Hospitalizations, SLE exacerbation	3 (33.3%)	22 (24.7%)	0.572
Hospitalizations, infection	2 (22.2%)	27 (30.3%)	0.611
Hospitalization, CVE	2 (22.2%)	14 (15.7%)	0.616
ESKD	0 (0.0%)	13 (12.6%)	0.257
Mortality	1 (11.1%)	7 (7.8%)	0.726

All comparisons are unadjusted. Values are presented as mean ± SD, or n (%), as appropriate

*SGLT2i* sodium–glucose transport 2 inhibitors, *ED* emergency department, *SLE* systemic lupus erythematosus, *CVE* cardiovascular event, *ESKD* end-stage kidney disease

### All-cause mortality

Mortality was similar in both groups [1 patient (11.1%) in SGLT2is-treated patients vs. 7 patients (7.8%) in controls, p = 0.7]. Kaplan–Meier survival analysis showed comparable outcomes between groups (log-rank test, p = 0.844). Consistent with this, a multivariable Cox model adjusted for age, sex, and the presence of CHF and DM demonstrated no significant difference in mortality between SGLT2is-treated patients and controls (data not shown).

## Discussion

This study is one of the first studies to describe real-life data examining the use of SGLT2is in patients with SLE. While extensive research has traditionally focused on immunosuppressive therapies, this study responds to the emerging need to address non-immunosuppressive interventions in SLE, potentially preventing damage and mortality as consequences of late complications of the disease, such as ESKD and cardiovascular disease [19, 22, 27, 28].

The use of SGLT2is in 9 SLE patients with DM [6], CKD [7], and cardiac disease [3] showed a trend of reduction in proteinuria, stable kidney function over-time, and without adverse cardiovascular events despite a high-risk population. Treatment was well-tolerated, with minimal side effects. Yet, a substantial number of patients with an indication for treatment with SGLT2is agents did not receive this treatment during data collection. This could be explained by the lack of evidence for the SLE population and the relatively new subsidy for its cost indication in CKD patients in Israel.

While the SGLT2is-treated patients were older and had higher comorbidities burden (including diabetes, chronic kidney disease, and heart failure), a similar rate of hospitalizations and cardiovascular events was observed in both SGLT2is-treated patients and controls. Regarding kidney function decline, due to the progressive nature of CKD, we observed eGFR decline in both SGLT2is-treated patients and controls, with a trend to stabilization of eGFR in SGLT2is-treated patients. We also notice a slight increase in serum creatinine 1 month after starting treatment, reflecting the dip phenomenon. Although having a small group of SGLT2is-treated patients and a short follow-up time, this trend is in keeping with the reno-protective effect of SGLT2is therapy. Furthermore, the trend toward a decrease in albuminuria in the SGLT2is-treated patients suggests a potentially positive impact on kidney function and is in line with the finding described by Caravaca-Fontan et al. [21].

There are data on the outcomes of SGLT2is treatment in non-diabetic CKD patients. In the Dapagliflozin and Prevention of Adverse Outcomes in Chronic Kidney Disease (DAPA–CKD) trial [17], at a median follow-up of 2.4 years, the SGLT2is, treated patients showed a reduction in all-cause mortality, ESKD, and the risk of 50% or greater decline in eGFR. The beneficial effect of dapagliflozin was similar in patients with and without diabetes as well as in patients with and without severe CKD. In the Study of Heart and Kidney Protection with Empagliflozin (EMPA–KIDNEY) trial, at 2 years, the SGLT2is showed a reduction in the incidence of ESKD, and the incidence of a sustained decrease in eGFR [18]. The risks of all-cause mortality and nonfatal cardiovascular events were comparable between the groups. Effects were similar in patients with and without diabetes regardless of the eGFR at the start of the study. The benefit from empagliflozin was greater in patients with an albumin-to-creatinine ratio of ≥ 300 mg/g and significantly less in those with lower albumin excretion. These data led EULAR, ACR, and KDIGO guidelines to encourage physicians to treat CKD patients with these agents regardless of their primary renal disease, including SLE patients, despite the fact that they were not adequately represented in these trials [12, 13, 26]. The 2024 ACR guidelines for the treatment and management of lupus nephritis recommend considering SGLT2is for stable LN patients with DM, CKD, moderate–high proteinuria, or heart failure, with cautious use in patients on high-dose immunosuppression due to the increased risk of UTI [26]. Morales et al. added treatment with empagliflozin to five patients with LN in chronic and stable treatment with immunosuppression and residual proteinuria. Within 8 weeks of starting treatment, the patients experienced a decrease of 49.9% in proteinuria with minimal change in glomerular filtration rate [22, 23]. The use of SGLT2is was associated with a significant decrease in proteinuria with reasonably stable eGFR in additional observations [21, 24, 28–30]. In terms of preserving and stabilizing eGFR, SGLT2is are particularly important in patients with class II/IV LN, but also in those without biopsy-proven histology, as they are also at risk of developing CKD as we previously demonstrated [31]. In a recent large retrospective real-world cohort study from the Israeli Clalit registry including 4,354 SLE patients, 295 were treated with SGLT2is. Among them, only 35% had LN, and treatment was associated with preservation of kidney function over 24 months compared with non-users, supporting their potential role in kidney protection in the entire SLE patient population, even in the absence of LN [32].

Potential adverse events of SGLT2is treatment include infections (especially vulvovaginal candidal infections and UTIs, including fatal pyelonephritis and necrotizing fasciitis), ketoacidosis, and increased fracture risk (33). We noticed 2 events of uncomplicated UTI that did not require hospitalization and did not interfere with SGLT2is treatment. In addition, we found a comparable rate of severe infections in both the SGLT2is-treated group and the control group, albeit a higher proportion of older patients with higher rates of comorbidities in the former. No events of ketoacidosis or fractures were recorded. One patient in the SGLT2is-treated group died from a disseminated zoster infection, but this was not likely associated with the SGLT2is treatment. No difference was found in hospitalizations for SLE exacerbations and mortality rates.

Regarding ESKD, causality cannot be made from this retrospective analysis. The likely reason for the absence of ESKD in the SGLT2is-treated group is the selection bias; during the study period, an eGFR below 20 ml/min/m2 was a contraindication for SGLT2is, regardless of the indication. Yet the high rate of ESKD in the control group, in line with the accepted rate in the literature, highlights the need for renal protective treatments in SLE patients.

This study has several substantial limitations, mainly due to its retrospective and observational nature and the small number of patients treated with SGLT2is. First, there were considerable differences in clinical characteristics between the group treated with SGLT2is and the control group, such as a trend toward older age in the SGLT2is-treated group, making it challenging to compare them directly. Second, the limited sample size of SGLT2is-treated patients and the relatively short follow-up time hinder the ability to draw definitive conclusions. In addition, while nearly, all SGLT2is-treated patients received concomitant RAAS blockade, RAAS exposure was not captured for controls, therefore, the observed benefit cannot be disentangled from the effect of concurrent RAAS inhibition. These limitations also affect the interpretation of the observed differences in eGFR trajectories between SGLT2is-treated patients and controls. These factors suggest that the results, while aligned with emerging guideline recommendations, should be interpreted with caution. Finally, the retrospective nature of this study limits the information available.

**Conclusion**

In this real-world retrospective cohort of patients with SLE, a substantial number of patients with an indication for treatment with SGLT2is agents did not receive this treatment. Treatment with SGLT2is was associated with stable kidney function over-time and a trend toward reduced proteinuria, without an observed increase in adverse cardiovascular events, severe infections, or mortality. These findings are consistent with the 2024 ACR guidelines, which recommend considering SGLT2is for stable LN patients with diabetes, CKD, moderate–high proteinuria, or heart failure. Nevertheless, interpretation of the findings is limited by the small treated cohort, baseline differences between groups, and the observational study design. Larger prospective, randomized studies are needed to confirm the nephro- and cardioprotective efficacy and safety of SGLT2is in patients with SLE, especially those with LN.

## Ethical approval

The study was conducted after receiving the approval of the Institutional Human Subjects Ethics Committee. All data were collected anonymously. All methods were carried out in accordance with the relevant guidelines and regulations.

## Data Availability

The data that support the findings of this study are available on request from the corresponding author. The data are not publicly available due to privacy or ethical restrictions.
